# Does cognitive/physical screening in an outpatient setting predict institutionalization after hip fracture?

**DOI:** 10.1186/s12891-016-1272-8

**Published:** 2016-10-22

**Authors:** Markus T. Hongisto, Maria Nuotio, Tiina Luukkaala, Olli Väistö, Harri K. Pihlajamäki

**Affiliations:** 1 Department of Orthopedics and Traumatology, Seinäjoki Central Hospital, Hanneksenrinne 7, Seinäjoki, 60220 Finland; 2 Department of Musculoskeletal Diseases, Tampere University Hospital, Teiskontie 35, Tampere, 33521 Finland; 3 Department of Geriatric Medicine, Seinäjoki Central Hospital, Hanneksenrinne 7, Seinäjoki, 60220 Finland; 4 Science Center, Pirkanmaa Hospital District, Biokatu 6, Tampere, 33520 Finland; 5 School of Health Sciences, University of Tampere, Terveystieteiden yksikkö, 33014 Tampereen yliopisto, Finland; 6 University of Tampere, Koskenalantie 16, Seinäjoki, Finland

**Keywords:** Hip fracture, IADL, MMSE, Living arrangements, Institutionalization, Rehabilitation

## Abstract

**Background:**

Institutionalization after hip fracture is a socio-economical burden. We examined the predictive value of Instrumental Activities of Daily Living (IADL) and Mini Mental State Examination (MMSE) for institutionalization after hip fracture to identify patients at risk for institutionalization.

**Methods:**

Fragility hip fracture patients ≥65 years of age (*n* = 584) were comprehensively examined at a geriatric outpatient clinic 4 to 6 months after surgery and followed 1 year postoperatively. A telephone interview with a structured inquiry was performed at 1, 4, and 12 months after hip fracture.

**Results:**

Age-adjusted univariate logistic regression analysis revealed that IADL and MMSE scores measured at the outpatient clinic were significantly associated with living arrangements 1 year after hip fracture. Multivariate logistic regression analysis established that institutionalization 1 year after hip fracture was significantly predicted by institutionalization at 4 months (odds ratio [OR] 16.26, 95 % confidence interval [CI] 7.37–35.86), IADL <5 (OR 12.96, 95 % CI 1.62–103.9), and MMSE <20 (OR 4.19, 95 % CI 1.82–9.66). A cut-off value of 5 was established for IADL with 100 % (95 % CI 96 %–100 %) sensitivity and 38 % (95 % CI 33 %–43 %) specificity and for MMSE, a cut-off value of 20 had 83 % (95 % CI 74 %–91 %) sensitivity and 65 % (95 % CI 60 %–70 %) specificity for institutionalization. During the time period from 4 to 12 months, 66 (11 %) patients changed living arrangements, and 36 (55 %) of these patients required more supportive accommodations.

**Conclusion:**

IADL and MMSE scores obtained 4 to 6 months after hospital discharge may be applicable for predicting institutionalization among fragility hip fracture patients ≥65 years of age at 1 year after hip fracture. An IADL score of ≥5 predicted the ability to remain in the community. Changes in living arrangements also often occur after 4 months.

## Background

Hip fracture is a devastating event for older people that leads to increased risk of death and disability [13, 18]. Only half of the survivors rehabilitate to the level of previous mobility and activities of daily living (ADL) [15]. The age-adjusted incidence of fall-induced hip fractures has been decreasing in Western countries, yet the total number of hip fractures will rise due to the rapid growth of the older population [10]. In addition, comorbidities among hip fracture patients have been increasing at least since 1986 [3]. Mortality is high within the first year after hip fracture, and the increase in mortality continues until 5 years after hip fracture [5, 12].

Although several comorbidities and predictive factors for survival following hip fracture have been reported, there have been few clinical studies, especially prospective studies, regarding the role of mobility, need for assistance, and living arrangements in hip fracture mortality and disability. Risk factors for institutionalized living arrangements have been reported: increased age, admission from a care facility, high number of medications, pre-injury dependence, male sex, dementia, and a lower pre-fracture level of ADL [4, 22, 23].

Assessment of survivor health condition is crucial for allocating public health care resources to patients at risk for institutionalization. The ideal clinical test for recognizing hip fracture patients at risk for institutionalization would be easy to conduct, reliable, and inexpensive, with excellent sensitivity or specificity. Optimal predictive tests could be carried out as soon as possible after hip fracture, because the rehabilitation program should begin as soon as possible after hip fracture surgery. Recovery after surgery differs comprehensively and clinical tests conducted within the first few weeks after surgery may have reliability problems, especially in patients with surgical complications or mental disorientation. Therefore, clinical tests performed a few months later to predict those hip fracture patients at risk of institutionalized living arrangements could be useful, especially in cases of previously independent patients. The Instrumental Activities of Daily Living (IADL) assessment and Mini Mental State Examination (MMSE) carried out 4 to 6 months after hip fracture are clinical tests that may predict living arrangements 1 year after hip fracture. The IADL assesses the complex skills needed to successfully live independently, such as the ability to prepare meals, use the telephone, manage medications, travel in the community, and perform housework and basic home maintenance [11]. The MMSE is a quantitative measure of cognitive status in adults. It can be used to screen or estimate the severity of cognitive impairment at a given time-point [6].

The purpose of the present study was to examine the IADL and MMSE, as part of a comprehensive outpatient assessment 4 to 6 months after hip fracture, as predictors of living arrangements 1 year after hip fracture.

## Methods

A prospective population-based observational cohort study of 1033 consecutive hospital admissions of patients aged ≥65 years with hip fracture was conducted during the study period between April 1, 2008, and May31, 2013. Only the first hip fracture in each patient during the follow-up period was included. Pathologic and periprosthetic fractures were excluded. The referral area for hip fracture patients was the Hospital District of Southern Ostrobothnia, Finland, which has a population of 193,977. Residents ≥65 years of age represent 21 % of the total population according to Official Statistics of Finland, a statutory electronic population register. All patients who sustained a hip fracture inside referral area were admitted and underwent surgery at Seinäjoki Central Hospital.

For the purpose of the study, patients who were considered institutionalized, such as living in a health care center hospital or a care home providing 24-h care at baseline were excluded from the study. Other living arrangements were defined as living independently in their own home, living in their own home with organized home care, or living in an assisted living accommodation. Data on deaths were obtained from the Official Cause of Death Statistics of Finland, which covers essentially 100 % of the deaths in Finland

**Fig. 1:**
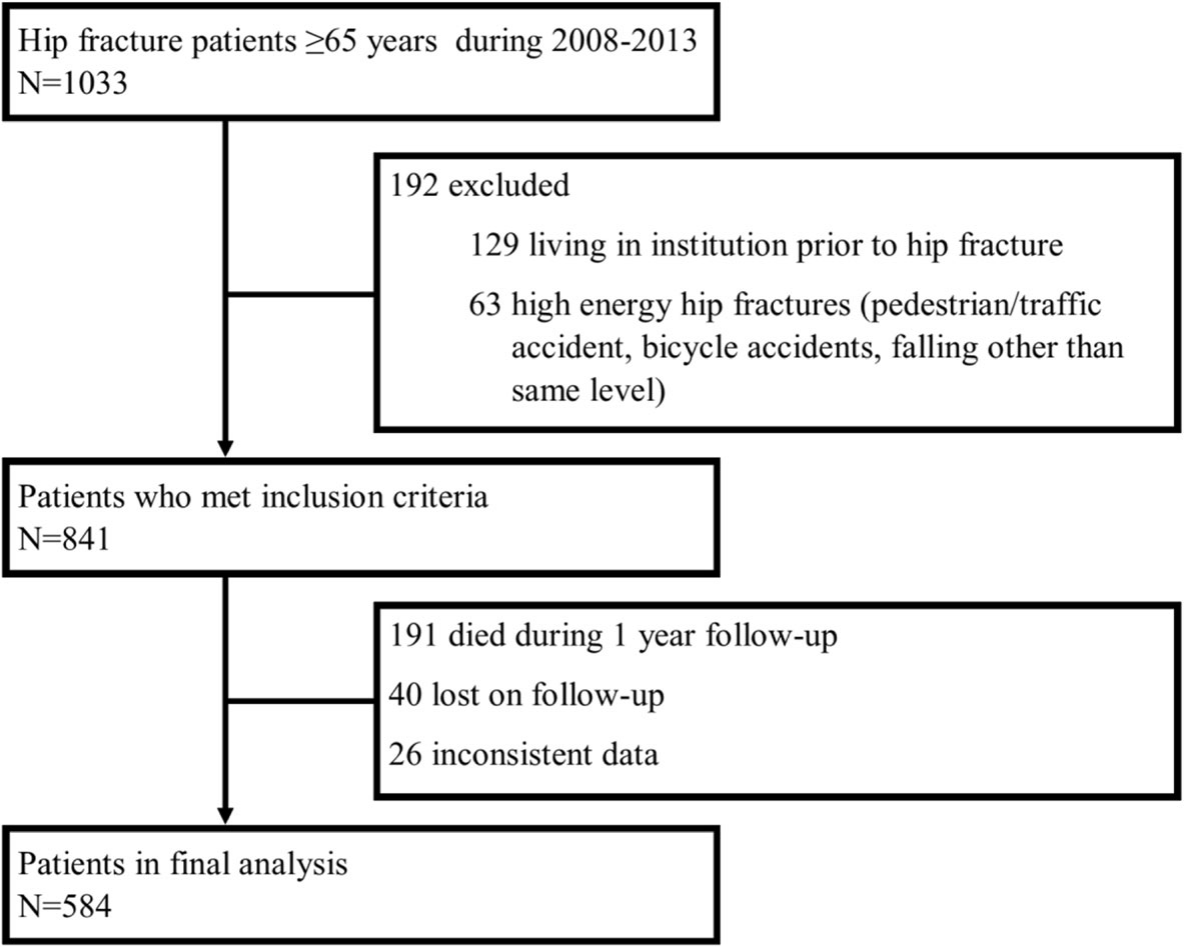
Study flow chart

The flow chart of the patient population is shown in Fig. 1. In all, 584 (70 %) patients completed the study with 12 months of follow-up and constituted the study population. The mean time from the hip fracture to an outpatient clinic visit was 5.1 (standard deviation 2.0) months with a median of 5 months (25–75 interquartile range: 4–6).

Patient information was collected using predefined inquiries and procedures on admission and a telephone interview was conducted by the same study nurse at 1, 4, and 12 months after surgery. To collect as accurate data as possible, we used data sheets modified from the British Hip Fracture Database [2]. If the patient was unable to provide the information, we used proxy respondents. Family members, friends, and nurses from an institution constituted the proxies. In addition, all patients, regardless of their place of residence, were invited to the geriatric outpatient clinic for comprehensive clinical assessment with a target time between 4 and 6 months after the fracture.

The primary outcome variable was living arrangements 1 year after hip fracture, which was categorized into two groups: not institutionalized (with or without organized home care in their own home or an assisted living accommodation) or institutionalized. IADL and MMSE performed at the outpatient visit were evaluated as predictor variables for living arrangements 1 year after the hip fracture. The Lawton-Brody IADL scale measures eight functional domains. IADL and MMSE were categorized in a dichotomous manner by using the best cut off value from the ROC analysis in this study, 5 and 20 respectively. Mobility aids were registered in the database as follows: independent, a cane, canes, folding or rollator walker, wheelchair, or immobile and bedbound. In this study, we categorized the need of mobility aids into mobile without an aid, mobile with an aid, or unable to ambulate. Further, the mobility level was classified as unassisted or assisted outdoors, unassisted or assisted indoors, and unable to move. Patients with an American Society of Anesthesiologists (ASA) grade of I or II were combined into one group because there were so few grade I patients (*n* = 3). Likewise, patients with an ASA grade of IV or V were combined because the number of patients with an ASA grade V, a moribund sub-population not expected to live 24-h with or without surgery, was also very small.

Statistical differences between categorical variables were calculated using Pearson’s chi-square test or Fisher’s exact. A *P*-value ≤0.05 was considered statistically significant. Institutionalized living arrangements 1 year after hip fracture were analyzed with age-adjusted univariate logistic regression analysis, and odds ratios (ORs) with 95 % confidence intervals (CI) for each variable were calculated. Multivariate logistic regression analysis using the enter (all variables included simultaneously into the model) method was used to investigate the independent effects of each statistically significant variable, except MMSE as measured at the outpatient clinic was used instead of previous diagnosis of memory disorder.

Receiver operating characteristics (ROC) analysis was used to compare the predictive power. The area under the curve (AUC) was calculated. A perfect model will score an AUC of 1, while random guessing will score an AUC of ~0.5. An AUC of 0.7 to 0.8 is considered to have good predictive power, that of 0.8 to 0.9 is considered to have excellent predictive power, and that >0.9 is considered to have outstanding predictive power. Sensitivity, specificity, positive (PPV) and negative (NPV) predictive values, and ORs were calculated with 95 % CI.

Survival analysis was conducted with age- and sex-adjusted Cox regression models to determine hazard ratios (HRs) for death 1 year after hip fracture. For this analysis, we used the study population (*n* = 841) that met the inclusion criteria. All statistical analyses were performed using SPSS version 21.

## Results

Mean patient age was 81.9 (SD 6.8) years, and 456 (78 %) of the 584 patients were women. In all, 380 (65 %) patients had a femoral neck fracture, 180 (31 %) had a pertrochanteric fracture, and 24 (4.1 %) a subtrochanteric fracture. Details of the baseline patient characteristics are provided in Table 1.

**Table 1:** Patient characteristics on admission (*n* = 841) and information of the 584 analyzed patients followed by baseline comparison between not institutionalized and institutionalized patients 1 year after hip fracture

			1 Year (*n* = 584)		
	Entire Cohort (*n* = 841)	Patients in Primary Analysis (*n* = 584)	Not institutionalized (*n* = 457)	Institutionalized (*n* = 127)	*P*-value
Variable	n (%)	n (%)	n (%)	n (%)	
Age					<0.001
65–74	117 (13.9)	87 (14.9)	81 (17.7)	6 (4.7)	
75–84	352 (41.9)	268 (45.9)	213 (46.6)	55 (43.3)	
> 85	372 (44.4)	229 (39.2)	163 (35.7)	66 (52.0)	
Mean (SD)	82.8 (7.1)	81.9 (6.77)	81.2 (6.8)	84.4 (6.1)	<0.001
Median (25–75 % percentile)	84.0 (78–88)	83.0 (77–87)	82.0 (76–86)	85.0 (81–88)	<0.001
Sex					0.352
Female	624 (74.2)	456 (78.1)	353 (77.2)	103 (81.1)	
Male	217 (25.8)	128 (21.9)	104 (22.8)	24 (18.9)	
Living with somebody					0.007
Yes	502 (59.7)	325 (55.7)	241 (52.7)	84 (66.1)	
No	339 (40.3)	259 (44.3)	216 (47.3)	43 (33.9)	
Mobility aids before hip fracture					0.012
Mobile without an aid	333 (39.6)	258 (44.2)	214 (46.8)	44 (34.6)	
Mobile with an aid	495 (58.9)	320 (54.8)	240 (52.5)	80 (63.0)	
Unable to ambulate	11 (1.3)	6 (1.0)	3 (0.7)	3 (2.4)	
Missing information	2 (0.2)				
Mobility level before hip fracture					<0.001
Unassisted outdoors	499 (59.3)	393 (67.3)	349 (76.4)	44 (34.6)	
Assisted outdoors	101 (12.0)	63 (10.8)	33 (7.2)	30 (23.6)	
Unassisted indoors	197 (23.4)	108 (18.5)	66 (14.4)	42 (33.1)	
Assisted indoors	28 (3.3)	13 (2.2)	6 (1.3)	7 (5.5)	
Unable to move	12 (1.4)	7 (1.2)	3 (0.7)	4 (3.1)	
Missing information	4 (0.5)				
Previous living arrangements					<0.001
Own home	390 (46.4)	293 (50.2)	263 (57.5)	30 (23.6)	
Own home with organized home care	265 (31.5)	193 (33.0)	141 (30.9)	52 (40.9)	
Assisted living accommodation	186 (22.1)	98 (16.8)	53 (11.6)	45 (35.4)	
Previous diagnosis of memory disorder					<0.001
Yes	180 (21.4)	120 (20.5)	63 (13.8)	57 (44.9)	
No	657 (78.1)	462 (79.1)	392 (86.2)	70 (55.1)	
Missing information	4 (0.5)	2 (0.3)			
Number of medications on admission					0.068
< 4	169 (20.1)	135 (23.1)	115 (25.2)	20 (15.7)	
4–10	531 (63.1)	366 (62.7)	281 (61.5)	85 (66.9)	
> 10	141 (16.8)	83 (14.2)	61 (13.3)	22 (17.3)	
Previous fracture of any bone					0.927
Yes	264 (31.4)	182 (31.2)	142 (31.1)	40 (31.5)	
No	576 (68.5)	402 (68.8)	315 (68.9)	87 (68.5)	
Missing information	1 (0.1)				
Hip fracture type					0.979
Femoral neck fracture	539 (64.1)	380 (65.1)	298 (65.2)	82 (64.6)	
Pertrochanteric fracture	259 (30.8)	180 (30.8)	140 (30.6)	40 (31.5)	
Subtrochanteric fracture	43 (5.1)	24 (4.1)	19 (4.2)	5 (3.9)	
ASA grade					0.002
1–2	114 (13.6)	95 (16.3)	87 (19.1)	8 (6.4)	
3	517 (61.5)	386 (66.1)	290 (63.7)	96 (76.8)	
4–5	197 (23.4)	99 (17.0)	78 (17.1)	21 (16.8)	
Missing information	13 (1.5)	4 (0.6)			

Institution represents hospital, health care center hospital, nursing home or rehabilitation unit providing 24-h care

Instrumental Activities of Daily Living (IADL), Mini Mental State Examination (MMSE), and American Society of Anesthesiologists (ASA) scores. Differences were tested for continuous age by Mann-Whitney U-test and median test. Categorical variables were tested by Pearson chi-square test or Fisher’s exact test

Age-adjusted univariate logistic regression analysis indicated that institutionalized living arrangements at 1 or 4 months, IADL and MMSE performed at the outpatient clinic, mobility level or living arrangements before fracture, living with somebody, ASA grade, age, and the number of medications on admission predicted living arrangements at 1 year after hip fracture (Table 2).

**Table 2:** Age adjusted univariate and multivariate logistic regression analysis demonstrating institutionalization at 1 year after hip fracture

		Age-adjusted univariate *n* = 584				Multivariate *n* = 472		
Variable	n	OR (95 % CI)		*P*	n	OR (95 % CI)		*P*
Living arrangements at 1 months								
Own home or assisted living accommodation	260	1.00			219	1.00		
Institution ^a^	324	3.81	(2.34–6.16)	<0.001	253	1.56	(0.67–3.63)	0.304
Living arrangements at 4 months								
Own home or assisted living accommodation	463	1.00			393	1.00		
Institution ^a^	121	33.24	(19.39–56.00)	<0.001	79	16.26	(7.37–35.86)	<0.001
IADL	487	2.54	(2.00–3.22)	<0.001				
5–8	199	1.00			197	1.00		
0–4	288	73.11	(10.03–532)	<0.001	275	12.96	(1.62–103.9)	0.016
MMSE		1.22	(1.16–1.27)	<0.001				
20–30	305	1.00			197	1.00		
0–19	180	9.00	(4.93–16.43)	<0.001	175	4.19	(1.82–9.66)	0.001
Age								
65–74	87	1.00			78	1.00		
75–85	268	3.49	(1.45–8.41)	0.005	221	1.12	(0.31–4.11)	0.865
> 85	229	5.47	(2.27–13.14)	<0.001	173	1.29	(0.35–4.71)	0.915
Mobility aids before fracture								
Mobile without an aid	258	1.00						
Mobile with an aid	320	1.23	(0.78–1.89)	0.360				
Unable to ambulate	6	5.03	(0.93–27.14)	0.061				
Mobility level before fracture								
Unassisted outdoors	393	1.00			343	1.00		
Assisted outdoors	63	6.18	(3.41–11.21)	<0.001	46	1.39	(0.53–3.65)	0.510
Unassisted indoors	108	4.27	(2.56–7.11)	<0.001	68	0.93	(0.37–2.34)	0.879
Assisted indoors	13	8.23	(2.61–25.98)	<0.001	8	0.96	(0.14–6.53)	0.968
Unable to move	7	12.04	(2.53–57.23)	0.002	7	0.89	(0.11–7.04)	0.914
Previous living arrangements								
Own home	298	1.00			256	1.00		
Own home with organized home care	193	2.74	(1.65–4.55)	<0.001	153	1.14	(0.48–2.76)	0.764
Assisted living accommodation	98	6.16	(3.51–10.81)	<0.001	63	1.17	(0.40–3.40)	0.777
Living with somebody								
Yes	325	1.00			253	1.00		
No	259	0.51	(0.34–0.78)	0.002	219	0.79	(0.33–1.87)	0.589
ASA grade								
1–2	95	1.00			79	1.00		
3	386	2.87	(1.32–6.20)	0.008	319	1.14	(0.33–3.97)	0.834
4–5	99	2.10	(0.86–5.12)	0.103	74	0.24	(0.05–1.13)	0.071
Medications on admission								
< 4 medicine	135	1.00			108	1.00		
4–10 medicine	366	1.76	(1.03–3.03)	0.040	297	1.01	(0.38–2.69)	0.991
> 10 medicine	83	2.06	(1.03–4.12)	0.041	67	1.72	(0.48–6.18)	0.406
Previous fracture of any bone								
Yes	182	1.00						
No	402	0.93	(0.60–1.43)	0.732				
Gender								
Female	456	1.00						
Male	128	0.94	(0.59–1.63)	0.871				
Hip fracture type								
Femoral neck fracture	380	1.00						
Pertrochanteric fracture	180	0.95	(0.61–1.47)	0.819				
Subtrochanteric fracture	24	1.05	(0.37–2.97)	0.930				

^a^Institution represents hospital, health care center hospital, nursing home, or rehabilitation unit providing 24-h care

Instrumental Activities of Daily Living (IADL), Mini Mental State Examination (MMSE), and American Society of Anesthesiologists (ASA) score. Results are shown as odds ratios (OR) with 95 % confidence intervals (CI). Statistically significant age adjusted variables from univariate logistic regression analysis were admitted to multivariate regression analysis

Multivariate logistic regression revealed institutionalized living arrangements at 4 months (OR 16.26, 95 % CI 7.39–35.86), IADL < 5 (OR 12.96, 95 % CI 1.62–103.9), and MMSE < 20 (OR 4.19, 95 % CI 1.82–9.66) were independently significant predictors for institutionalization (Table 2).

**Fig. 2:**
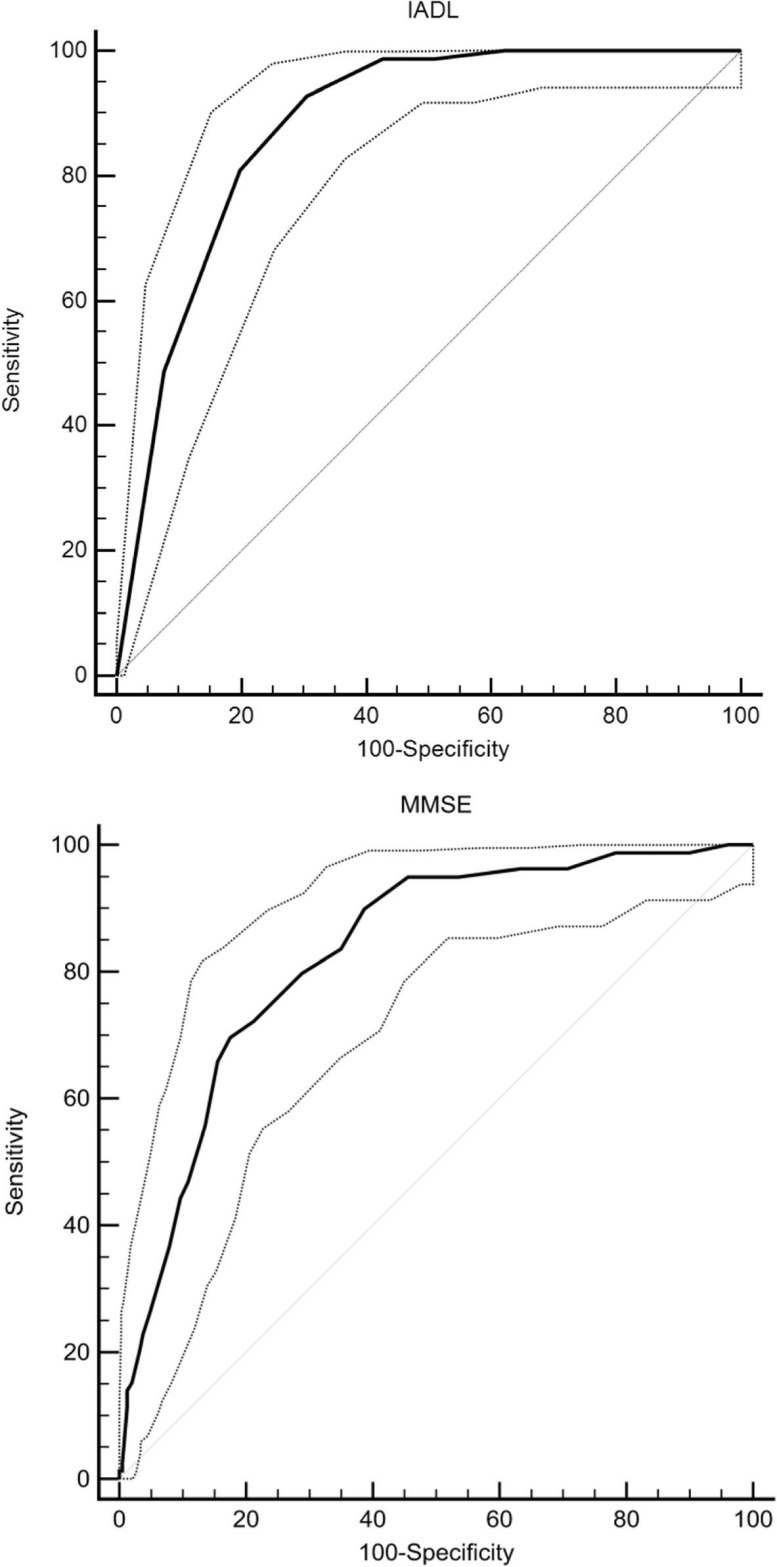
ROC curves for IADL and MMSE with 95 % confidence interval

ROC analysis revealed excellent discrimination for the IADL (0.88, 95 % CI 0.85–0.91) and MMSE (0.83, 95 % CI 0.79–0.86; Fig. 2). With regard to institutionalization, a cut-off value of 5 was established for IADL with 100 % (95 % CI96%–100 %) sensitivity and 38 % (95 % CI 33 %–43 %) specificity, which lead to a PPV of 0.251 and NPV of 1.00. The OR could not be calculated, because no patient with an IADL score of ≥5 was institutionalized when the 95 % CI was used. For the MMSE, a cut-off value of 20 had 84 % (95 % CI 74 %–91 %) sensitivity and 65 % (95 % CI 60 %–70 %) specificity with a PPV of 0.317 and an NPV of 0.953. An OR of 9.4 (95 % CI 5.0–17.7) was determined for institutionalization. Alternative cut-off values with detailed statistical information are provided in Table 3.

**Table 3:** Receiver-operating characteristics (ROC) analysis. Cut-off values, sensitivity, specificity, positive predictive value (PPV), negative predictive value (NPV), and odds ratios (ORs) with 95 % confidence intervals (95 % CI) for Instrumental Activities of Daily Living (IADL) and Mini Mental State Examination (MMSE) for predicting institutionalized living arrangement at 1 year after fragility hip fracture

Cut-offs	Sensitivity	Specificity	PPV	NPV	OR	(95 % CI)
IADL						
2	92.9 %	69.5 %	0.388	0.979	29.6 (12.6–69.7)	
3	98.8 %	57.3 %	0.325	0.996	111.5 (15.4–808.7)	
4	98.8 %	49.1 %	0.288	0.995	80.2 (11.1–581.5)	
5	100 %	38.0 %	0.251	1.000	Undefined	
MMSE						
10	22.8 %	96.3 %	0.293	0.865	7.7 (3.7–16.1)	
15	55.7 %	86.5 %	0.444	0.909	8.0 (4.7–13.6)	
20	83.5 %	65.0 %	0.317	0.953	9.4 (5.0–17.7)	
25	96.2 %	29.3 %	0.209	0.975	10.5 (3.3–34.0)	

Overall mortality during the 12-month follow up was 23 % (*n* = 191). The highest proportional mortality 62 % (*n* = 119) was observed within the first 3 months, followed by 16 % (*n* = 31) proportional mortality between 3 to 6 months after hip fracture. During the 6 to 9 months and 9 to 12 months after hip fracture, the proportional mortality was 7.9 % (*n* = 15) and 14 % (*n* = 26), respectively. Age- and sex-adjusted Cox regression models showed that institutionalization at 1 (HR 2.28, 95 % CI 1.47–3.54) and 4 (HR 3.50, 95 % CI 2.00–6.11) months after hip fracture considerably increased the HR for death 12 months after hip fracture.

**Fig. 3:**
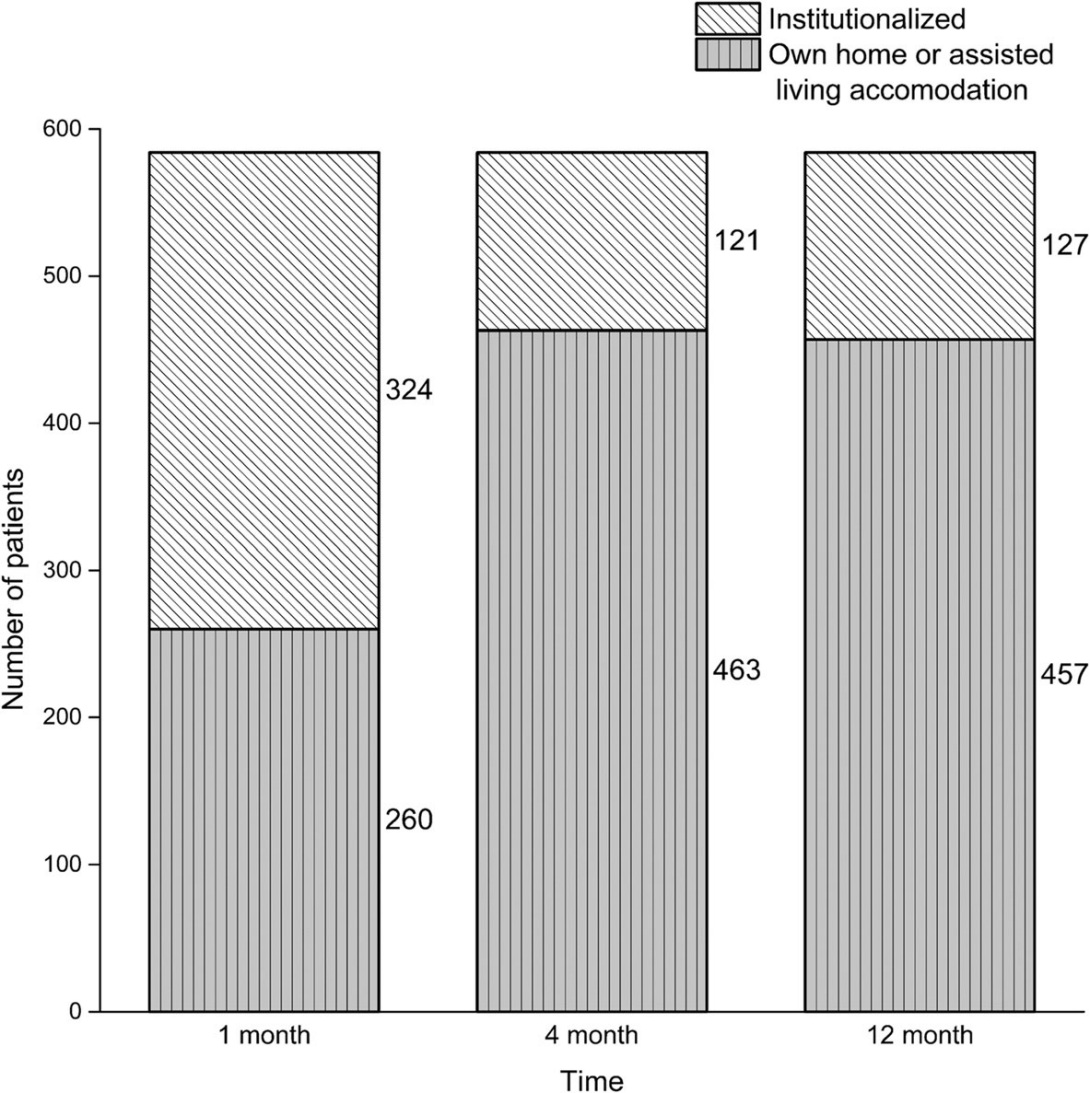
The absolute number of patients with different living arrangements at 1, 4, and 12 months after hip fracture. Institutionalized represents hospital, health care center hospital, nursing home, or rehabilitation unit providing 24-h care

**Fig. 4:**
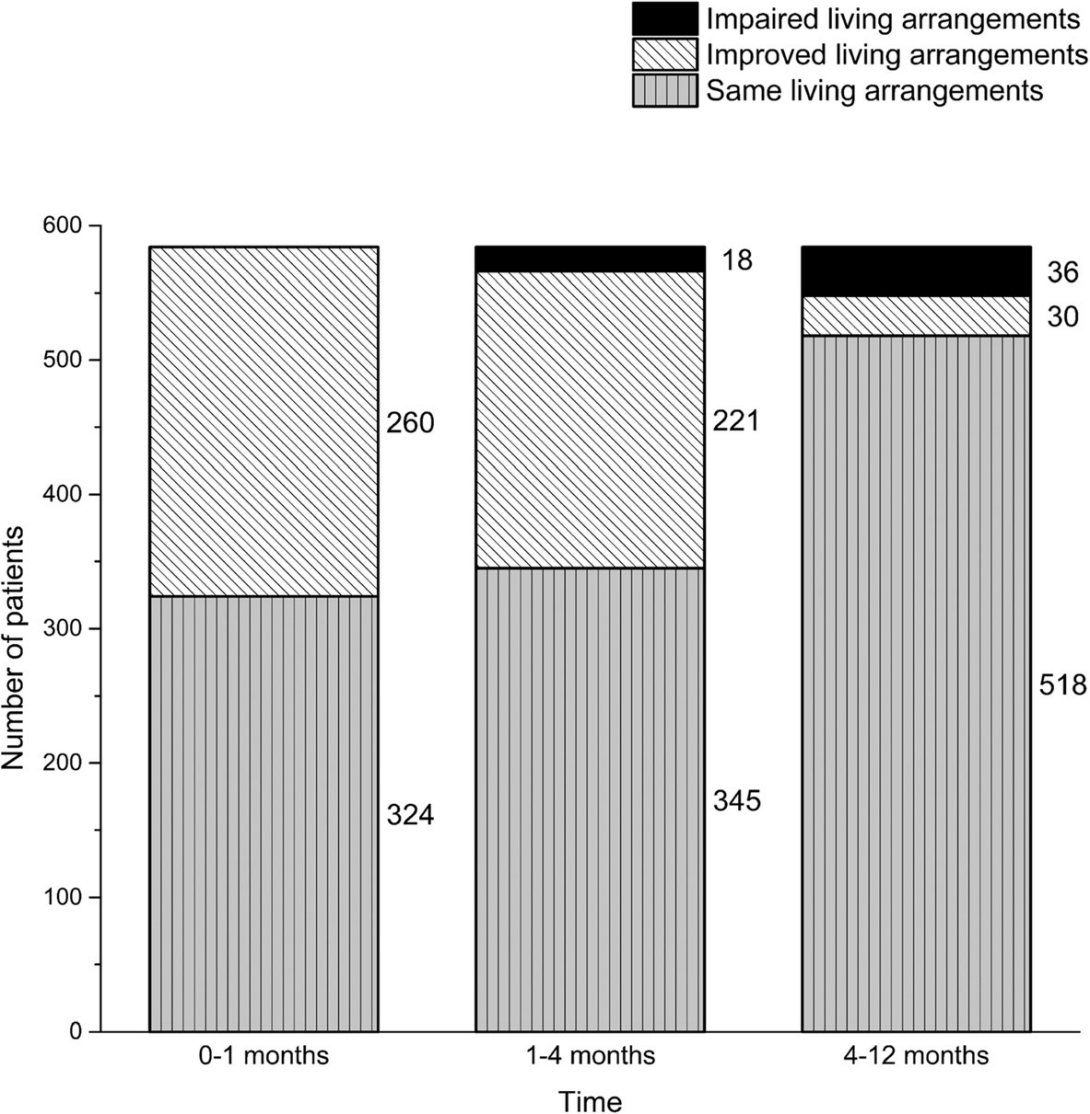
Change in living arrangements between hospital discharge and 1, 4, and 12 months

The living arrangements were observed at 1, 4, and 12 months after hip fracture (Fig. 3). Changes in the living arrangements are shown in Fig. 4. One month after hip fracture, 324 (56 %) were institutionalized, of which 221 (68 %) had improved living arrangements at 4 months. Of the 260 patients living in their own home or in an assisted living accommodation prior to the hip fracture, 18 (6.9 %) were institutionalized at 4 months. Of the 121 patients institutionalized at 4 months, 30 (25 %) were able to live on their own or in an assisted living accommodation by 12 months. Of the 463 patients who were not institutionalized at 4 months, 36 (7.8 %) were institutionalized by 12 months. All changes in living arrangements were statistically significant. A total of 66 (11 %) patients changed their living arrangement during between 4 and 12 months after hip fracture.

## Discussion

Our findings revealed that IADL and MMSE performed 4 to 6 months after hip fracture in older patients independently predicted institutionalized living arrangements 1 year after hip fracture. Further, an IADL cut-off value of ≥5 provided 100 % sensitivity and 38 % specificity for institutionalization. Thus, the IADL results identified patients at risk for institutionalized living. Mortality after hip fracture surgery was highest during the first 3 postoperative months and patients living in an institution 1 or 4 months after hip fracture had a higher HR for death. Further, rehabilitation occurred mostly within the first 4 months, and thereafter the cumulative changes in the living arrangements were minor.

We focused on finding statistically significant variables and cut-off values for older hip fracture patients at risk for institutionalization. A previous study reported significant improvement in IADL abilities between 3 months and 1 year after hip fracture [14]. On the other hand, a 1-year longitudinal study with 225 community residents aged ≥65 showed functional improvement at 2 months following post-acute rehabilitation with continued improvement up to 6 months, after which functional recovery slowed and remained constant through 12 months [24]. That study population, however, comprised patients with only subcapital hip fractures and the recovery patterns were heterogeneous, indicating that the study results cannot be generalized to all hip fracture patients. Heikkinen et al examined 196 consecutive hip fracture patients aged ≥50 years to compare functional outcome at 4 and 12 months after hip fracture. They concluded that a 4-month follow-up is the shortest possible period, because living arrangements and most functional outcomes do not change significantly after 4 months [8]. Our findings were similar within the first 4 months, but contradict the change in residential location thereafter; in our study population, 66 (11 %) changed living arrangements between 4 and 12 months.

The MMSE is the most commonly used instrument for screening cognitive function. Hip fracture is more common in older people with cognitive impairment, and hip fracture patients with cognitive impairment, including mild to moderate dementia benefit from postoperative geriatric rehabilitation [1, 16, 17, 20]. Further, a lower MMSE score increases the fall risk [7, 19]. In a randomized control trial, 173 patients with mild or moderate cognitive impairment (MMSE range 15–25) had a more than 7-fold increased risk for nursing home admission in the first year after hip fracture [21]. Our results are consistent with this finding when we applied a cut-off value of 20. Education level affects the MMSE score; a highly educated person with mild cognitive impairment may have a normal MMSE score, whereas a patient with less education will have a subnormal MMSE score [9]. ROC analysis established excellent discrimination for the MMSE and a cut-off value of 20 indicated strong (84 %) sensitivity, but only fair (65 %) specificity, with an OR 9.4 for institutionalization. With this cut-off value, the MMSE failed to predict institutionalization for 17 % of patients, but falsely predicted institutionalized living arrangements for 35 % of patients. Thus, setting optimal cut-off values remains controversial, though in the multivariate logistic regression analysis lower MMSE scores predicted institutionalization at 12 months. Increasing the cut-off value would increase false positives and decrease the true negative test results for institutionalization. Therefore, the ideal cut-off value cannot be confirmed.

Some baseline characteristics and clinical tests in the univariate analysis also predicted institutionalization, although they were inferior compared to the IADL and MMSE overall. Unexpectedly, the need for ambulatory aids before hip fracture did not predict institutionalization after adjusting for age (Table 2). Notably, only six patients among the patients who completed the study were unable to ambulate before hip fracture, and for this group the *p*-value for institutionalization was 0.061. Thus, according to our study, the need for ambulatory aids before fragility hip fracture did not markedly affect the living accommodations of hip fracture patients with the exception of immobile patients who had a moderate risk for more supported living arrangements in the future.

Chronologic age appeared to have a significant effect on living arrangements 1 year after hip fracture, but after adjusting for confounders, the effect of was no longer statistically significant. We believe that patients with several co-morbidities and impaired functional status prior to hip fracture are more likely to die within the first year after an accident. Thus, we suggest that survivors represent a sub-population younger in biologic age and in better health, which reduced the effects of increased chronologic age.

After adjusting for confounders, institutionalized living arrangements 1 month after hip fracture, in contrast to the 4-month living arrangements, did not predict institutionalization at 1 year after hip fracture. We conclude that rehabilitation after hip fracture proceeds favorably for at least first 4 months, but thereafter the recovery rate decreases and the risk for less independent living arrangements and death is increased. Therefore, we recommend that the most intensive rehabilitation continue for at least the first 4 months after hip fracture and then special attention should be focused on patients with known risk factors for institutionalization to avoid future institutionalized living arrangements.

This study has some limitations: 1) Dependence on data reported by patients or proxies, which might lead to under- or overestimation of patient mobility and living facilities; 2) Although we used pre-defined inquiries for the data collection, we could build a multivariate logistic regression model for only 472 (81 %) patients due to inconsistent data; 3) Living arrangements 1 and 4 months after hip fracture provide information only about institutionalization, and long-term care and rehabilitation were not differentiated; 4) Living arrangements and rehabilitation regimens after hip fracture differ greatly among countries, and the study results may not be universal. A major strength of the study was that the research material represented a population-based sample of older hip fracture patients. Finally, only 40 (4.8 %) patients were lost to follow-up and all patients inside the referral area were admitted and operated on at Seinäjoki Central Hospital, instead of multiple centers, which could lead to different surgical techniques and implant usage as well as different rehabilitation programs.

## Conclusion

IADL and MMSE tests performed in fragility hip fracture patients ≥65 years of age 4 to 6 months after hospital discharge predicted institutionalization at 1 year after hip fracture. An IADL score of ≥5 predicted the ability to remain in the community. Changes in residential location occurred mainly within the first 4 months, but changes in living arrangements were also observed from 4 to 12 months, indicating the need for screening methods to detect hip fracture patients at greater risk of institutionalization.

## Abbreviations

ADL: Activities of daily living
ASA-grade: American society of anesthesiologists grade
AUC: Area under the curve
HR: Hazard ratio
IADL: Instrumental activities of daily living
MMSE: Mini mental state examination
NPV: Negative predictive value
PPV: Positive predictive value
ROC: Receiver operating characteristics
